# Association between symptom experience and long-term survival in people treated for cancer

**DOI:** 10.1016/j.esmoop.2026.108579

**Published:** 2026-09-21

**Authors:** K. Hofsø, T. Rustøen, K. Bjordal, M. Hagen, B. Aouizerat, G.L. Astrup

**Affiliations:** 1 Department of Postoperative and Critical Care Nursing, Division of Emergencies and Critical Care, Oslo University Hospital, Oslo, Norway; 2 Department for Postgraduate Studies, Lovisenberg Diaconal University College, Oslo, Norway; 3 Department of Public Health and Interdisciplinary Health Science, Institute of Health and Society, Faculty of Medicine, University of Oslo, Oslo, Norway; 4 Department of Research and Development, Division of Emergencies and Critical Care, Oslo University Hospital, Oslo, Norway; 5 Research Support Services, Oslo University Hospital, Oslo, Norway; 6 Institute of Clinical Medicine, Faculty of Medicine, University of Oslo, Oslo, Norway; 7 Department of Nursing and Health Promotion, Faculty of Health Sciences, Oslo Metropolitan University, Oslo, Norway; 8 Department of Maxillofacial Surgery, College of Dentistry, New York University, New York, USA; 9 Translational Research Center, College of Dentistry, New York University, New York, USA; 10 Department of Oncology, Division of Cancer Medicine, Oslo University Hospital, Oslo, Norway

**Keywords:** cancer, patient-reported outcomes, survival, symptoms

## Abstract

**Background:**

Both symptoms and other aspects of health-related quality of life have been shown to be prognostic factors for cancer survival. Less is known about the association between distinct symptom subgroups and survival. Thus, this prospective study aimed to explore the possible association between symptom class membership during cancer treatment and long-term all-cause mortality, adjusting for known sociodemographic and clinical prognostic factors.

**Patients and methods:**

Between 2008 and 2011, 534 patients with cancer were included in a study assessing self-reported symptoms during cancer treatment. Symptom class membership before initiation of new treatment was investigated and four subgroups were identified on the basis of the occurrence of symptoms [i.e. ‘all low’ (28% of all patients), ‘all high’ (28%), ‘high psychological’ (23%), and ‘low psychological’ (21%)]. In the present follow-up analysis, multivariate Cox proportional hazards regression models were used to estimate the strength of a possible association between symptom class membership and all-cause mortality by comparing the ‘all high’ subgroup with the three other subgroups 12-15 years after study inclusion.

**Results:**

When adjusted for sociodemographic and clinical variables (i.e. sex, age, cancer diagnosis, and comorbidities), belonging to the ‘all high’ subgroup compared with the three other subgroups was associated with significantly higher mortality (hazard ratio 1.48, 95% confidence interval 1.14-1.94).

**Conclusion:**

The present study identified an association between symptom experience and long-term survival. This finding suggests that symptom class membership may be an important prognostic marker for identifying patients at increased risk of all-cause mortality.

## Highlights

• Patients with multiple symptoms at the start of a new cancer therapy had an increased mortality risk 12-15 years later.
• The risk is increased regardless of known prognostic factors such as age, sex, comorbidities, and type of cancer diagnosis.
• Men had an increased mortality risk compared with women.

## Introduction

The leading cause of death in Norway in the previous years was cancer, with a relative 5-year survival of 78% for both men and women with cancer in 2024.^1^ The type and stage of the cancer diagnosed, the available treatment, and the timing of initiation of treatment after diagnosis contribute to varying survival rates.^2^ Comorbidities, health-related lifestyle behaviors, and sociodemographic factors may also affect survival.^3^

Previous studies among different patient populations with cancer have identified distinct subgroups or classes of patients with different symptom burdens (e.g. low, moderate, or high) on the basis of self-report of the occurrence, frequency, severity, or distress of multiple symptoms.^4^, ^5^, ^6^, ^7^, ^8^, ^9^, ^10^, ^11^, ^12^, ^13^ A higher symptom burden has been associated with younger age,^7^^,^^14^^,^^15^ being female,^15^ a lower level of education,^10^ being unemployed,^7^^,^^12^ having a lower income,^12^^,^^15^ and not being married.^12^^,^^14^ In addition, several studies have found associations between higher symptom burden and more comorbidities^7^^,^^8^^,^^10^^,^^12^ and poorer functional status^7^^,^^8^ in various patient populations with cancer.

Similar results were found in our previous work, in which we identified latent symptom classes in patients with breast, head and neck, colorectal, or ovarian cancer (*N* = 534) on the basis of the probability of symptom occurrence.^13^ Symptoms were assessed before the start of a new treatment [i.e. radiotherapy (RT) or chemotherapy (CTX)] and patients were classified into four latent classes using latent class analysis (LCA): ‘all low’ (28% of all patients), ‘all high’ (28%), ‘high psychological’ (23%), and ‘low psychological’ (21%).^13^ Patients in the ‘all high’ class were younger than patients in the ‘all low’ class and had lower functional status, had more comorbidities, and reported poorer health-related quality of life (HRQoL) scores compared with the patients in the remaining symptom classes. Although no other statistically significant differences were found, slightly more patients in the ‘all high’ class had metastases (i.e. ≥1 versus none) and received treatment with palliative intent (i.e. versus curative) compared with patients in the other classes. Moreover, the distribution of cancer diagnoses varied somewhat (e.g. more patients with head and neck cancer in the ‘all high’ class than in the other classes).

The association between patient-reported outcomes (PROs) and survival in patients with cancer has been investigated for some time,^16^, ^17^, ^18^, ^19^ most commonly using global quality of life (QoL) scales or functional scales of HRQoL instruments. Recent publications of a pooled analysis of registry data^20^ using a summary score (i.e. global QoL and physical functioning scale), a meta-analysis using global QoL,^21^ and a meta-analysis using different PRO domains^22^ found that worse results on several measures were associated with higher mortality risk. In these various cancer populations, worse global QoL was associated with higher mortality risk before and after treatment and in the palliative phase. However, it is not entirely clear which HRQoL domains are the best prognosticators for mortality. Several symptom scales have also been found to have prognostic value,^22^^,^^23^ such as the Edmonton Symptom Assessment System^15^^,^^24^ and the Memorial Symptom Assessment Scale (MSAS).^25^ The recent publication by Huang et al.^22^ demonstrated compelling associations between symptom burden and mortality; however, the association between overall symptom experience or symptom class membership and long-term survival is less frequently studied. An example is a recent study using LCA to identify subgroups of patients with different physical well-being profiles after hematopoietic stem cell transplantation. This study found that 5-year survival differed among the symptom classes.^26^ Another example is a study of older patients with cancer using LCA to identify subgroups with different depressive symptom profiles that found that severe depression was associated with poorer 3-year survival.^6^ To our knowledge, no studies have investigated the association between symptoms and long-term (>5 years) survival, a recognized minimum threshold for survival studies. Therefore, more research is needed to better understand the prognostic importance of self-reported symptoms for long-term survival in patients with cancer. Thus, the purpose of the present study was to explore the potential association between the level of symptom burden at initiation of new cancer treatment and long-term survival.

## Methods

### Patients and settings

This longitudinal study evaluated symptoms and HRQoL outcomes in 534 patients with cancer and study procedures are described in detail elsewhere.^13^ Patients were enrolled between December 2008 and February 2011. Briefly, patients were included if they were adults (≥18 years of age); able to read, write, and understand Norwegian; and scheduled to receive RT for breast or head and neck cancer or CTX for colorectal or ovarian cancer. They were excluded if they were scheduled to receive RT for brain metastases or had a disease that affected their cognitive ability. Patients who did not complete PRO measures were excluded from the analyses presented in this article. Patients were recruited before initiation of treatment at Oslo University Hospital in Norway.

### The Outcome

The main outcome was patient survival, defined as the interval from the date of enrollment to the date of death (all-cause mortality) or the end of study (censor date 5 May 2023), whichever came first.

### Data collection

Data were collected through self-report from the patients and from the medical records. All patients completed the PRO measures described below at enrollment and at selected time points until 6 months after inclusion. Only data from the first symptom assessment (before initiation of new treatment) were used in the analyses for the present study.

### Demographic and clinical variables

Patients provided information on marital status, living situation, and level of education. Study nurses obtained information on sex, age, the presence and number of metastatic sites if applicable, intention of the treatment (i.e. curative or palliative), and the number and types of previous treatments from the patients’ medical record.

### Performance status

The Karnofsky Performance Status (KPS) scale^27^ was used to rate the patients’ self-reported functional status; the scale ranged from 0 (death) to 100 (normal; no complaints; no evidence of disease). The KPS scale has established its validity and reliability in similar study populations.^28^ Because patients in this study were outpatients at enrollment, only the 40 (disabled, need special help and care) to 100 point range of the KPS scale was used.

### Comorbidities

The Self-Administered Comorbidity Questionnaire (SCQ)^29^ was used to report whether patients had 16 common and three optional medical conditions (yes/no), if they received treatment for them (yes/no), and if the condition limited their activities (yes/no). The total SCQ score can range from 0 to 57; a higher total score indicates a more severe/higher level of comorbidity. The SCQ has established its validity and reliability in patients with chronic medical conditions.^29^ In the present study, only the total number of comorbidities (0-19) was used in the statistical analyses.

### Multiple symptoms

Data on symptoms were collected using MSAS,^30^ which consists of 32 physical and psychological symptoms that can occur during the cancer disease and its treatment. Patients indicated whether they had the symptoms during the past week (i.e. occurrence), and if they did, they rated its frequency (1-4), severity (1-4), and distress (0-4) on a Likert scale. Higher scores indicated a more frequent, severe, or distressing symptom experience. If a patient rated the frequency, severity, or distress of a symptom as >0 despite reporting ‘no’ on symptom occurrence, occurrence was coded to indicate that the patient had the symptom. All missing single items were interpreted and coded assuming that the patient did not have the symptom. The occurrence rates for the 32 symptoms were summarized and used to quantify the total number of symptoms each patient experienced. The MSAS has well-established reliability and validity in various cancer populations.^30^ For the present study, the occurrences (yes/no) were used to identify subgroups of patients (i.e. latent classes) with distinct symptom experience.

### Statistical analyses

The data are described using medians and ranges for continuous variables and counts and proportions for categorical data. Identification of the latent classes is described in detail in a previous publication.^13^ In brief, LCA is a method for identifying unmeasured class membership among participants using observed variables and was used to identify subgroups of patients (i.e. latent classes) with distinct symptom experiences.^31^^,^^32^ To ensure a sufficient number of patients with each symptom, e.g. to ensure we had enough statistical power to perform the LCA, the analysis was conducted using symptom occurrence ratings for the 13 symptoms that occurred in ≥30% of the patients (i.e. lack of energy, feeling drowsy, pain, difficulty sleeping, dry mouth, problems with sexual interest/activity, feeling bloated, sweats, worrying, feeling sad, difficulty concentrating, feeling nervous, and feeling irritable). The LCA identified four classes of patients on the basis of the probability of the occurrence of these symptoms (Bayesian Information Criterion [BIC] 7973.14 and entropy 0.81 versus BIC >8000 and entropy 0.83 for the two-, three-, and five-class models).^13^ Members of the ‘all low’ class (*n* = 152) reported a relatively low likelihood of occurrence of all 13 symptoms; the ‘all high’ class members (*n* = 149) reported a relatively high likelihood of occurrence of all 13 symptoms; the ‘high psychological’ class members (*n* = 121) reported a low-to-moderate likelihood of occurrence of the physical symptoms and a higher likelihood of occurrence of the psychological symptoms; and finally, the ‘low psychological’ class members (*n* = 112) reported a moderate-to-high likelihood of occurrence of the physical symptoms and a lower likelihood of occurrence of the psychological symptoms.

The crude cumulative mortality for each class was depicted using the Kaplan–Meier method. Years from enrollment to death (event) or 5 May 2023 (censoring) constituted the time of analysis. The latent class with the largest class size (i.e. ‘all low’) was selected as the reference class. To assess the strength of a possible association between class membership and overall mortality, we fitted Cox proportional hazards models. Two models were fitted: one using the four latent classes previously identified and one using the ‘all high’ class versus the remaining three classes. The models were adjusted for age, sex, level of education, cancer diagnosis, and number of comorbidities, selected on the basis of previous empirical data. Functional status, commonly associated with survival, was omitted owing to the very skewed distribution of the KPS scores. We divided the possible predictive factors (here treated as confounders) into blocks and entered each block separately into a Cox model. In step 2, we entered class membership and variables statistically significantly associated with the outcome from step 1. The results are presented as hazard ratios (HRs) with 95% confidence intervals (CIs). The proportional hazards assumption of the Cox model was tested by visual inspection of Kaplan–Meier curves and Schoenfeld’s residuals. Sensitivity analyses were carried out by repeating the Cox proportional hazards models using only complete cases for MSAS (i.e. excluding patients with missing single items for any symptom). Analyses were carried out using Stata version 18 (StataCorp, College Station, TX). A *P* value of 0.05 was considered statistically significant for all analyses. All analyses were considered exploratory, so no correction for multiple testing was carried out.

## Results

### Patient characteristics

Demographic and clinical characteristics of the sample are presented in Table 1. Among the 534 patients enrolled in the study, the median age was 61 years (min-max 23-87 years) at the time of enrollment and 367 (69%) were women. There were 188 patients with breast cancer, 133 with head and neck cancer, 120 with colorectal cancer, and 93 with ovarian cancer. The majority (72%) were treated with curative intent.

**Table 1:** Demographic and clinical characteristics of the sample at enrollment (*N* = 534)

Characteristic	Values
Age (years)	61 (23-87)
Number of comorbidities (0-19)^a^	1 (0-8)
Karnofsky Performance Status score (40-100)^b^	90 (40-100)
Sex	
Male	167 (31)
Female	367 (69)
Treatment intent	
Curative	387 (72)
Palliative	147 (28)
Diagnosis	
Breast	188 (35)
Head and neck	133 (25)
Colorectal	120 (23)
Ovarian	93 (17)
Level of education	
Primary	97 (18)
Secondary	246 (46)
College/University	182 (34)
Missing	9 (2)

Data are presented as median (min-max) or *n* (%).

a Missing *n* = 18.

b Missing *n* = 44.

### Survival by latent classes

Information on survival could not be retrieved for one patient from the ‘high psychological’ class. Among the 533 remaining patients, the median cumulative survival from study enrollment was 11.8 years (95% CI 9.9-13.1), varying from 7.6 years (95% CI 5.7-12.3) among patients in the ‘all high’ class to 12.9 years (95% CI 10.5-13.4) among patients in the three combined classes. As depicted with the Kaplan–Meier curves (Figure 1), a higher proportion of patients died in the ‘all high’ class, and after 15 years, 37% were alive in this group versus 48% being alive in the three combined classes.

**Figure 1:**
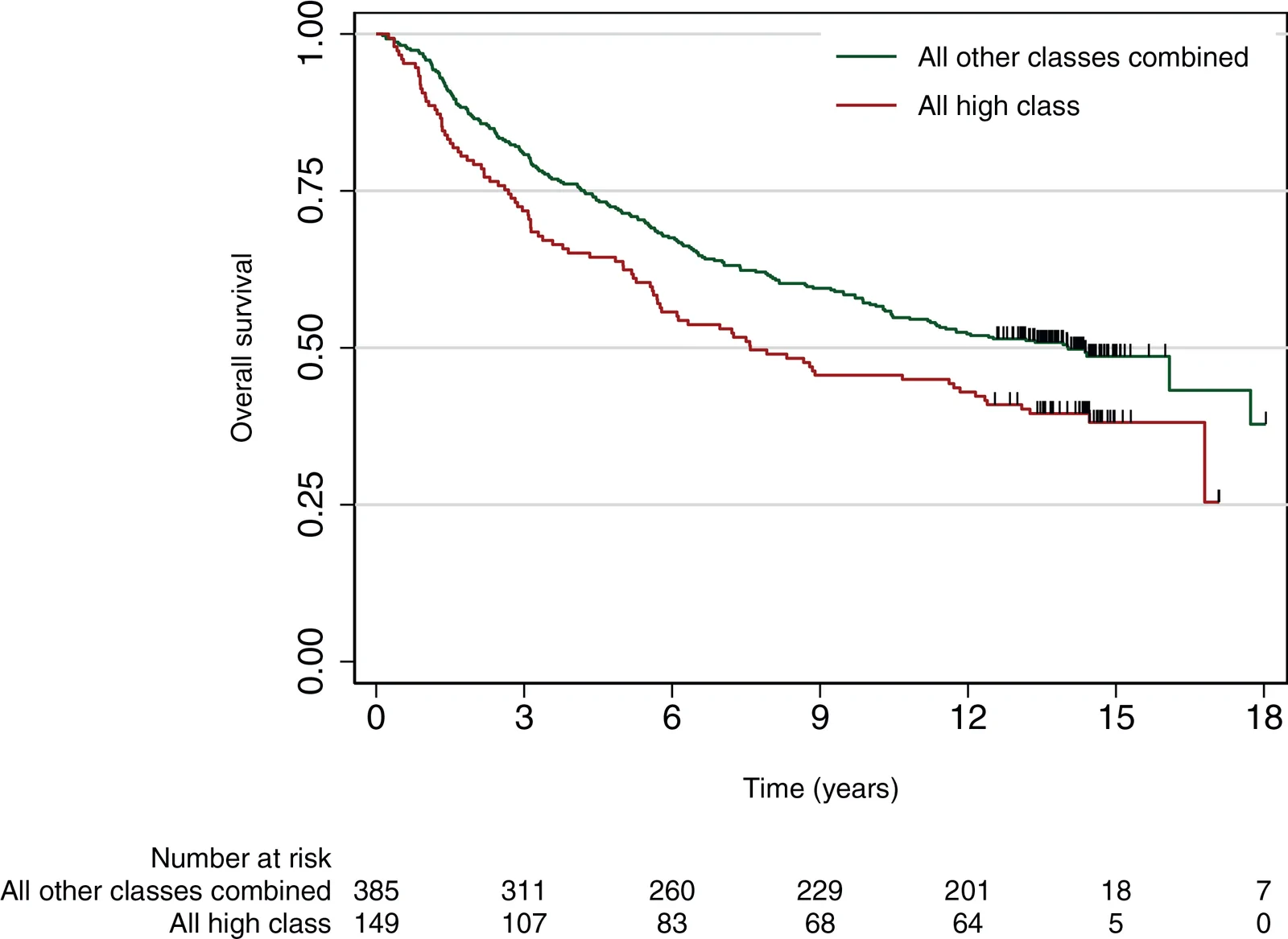
**Survival by symptom class.** Survival by symptom class membership for the ‘All high’ class versus the three other classes combined (i.e. ‘All low’, ‘High psychological’, and ‘Low psychological’).

The Cox proportional hazards models produced similar results when fitted with a four-class (Table 2) and a two-class (i.e. ‘all high’ versus the other three classes combined; Table 3) model. We will therefore focus on the two-class model because the reduction in number of symptom groups enabled us to derive estimates with higher precision. In univariate analyses, symptom class, sex, age, cancer diagnosis, and number of comorbidities were all statistically significantly associated with mortality; however, level of education was not (Table 3). In the multivariate analysis, when adjusted for sex, age, type of cancer diagnosis, and comorbidities, the patients in the ‘all high’ class were 48% more likely to die than patients in the other classes combined (HR 1.48, 95% CI 1.14-1.94; Table 3). The analysis also showed that men were 46% more likely to die than women and that higher age (HR 1.03, 95% CI 1.02-1.04) and higher number of comorbidities (HR 1.08, 95% CI 1.00-1.16) were both associated with an increased mortality risk. Compared with breast cancer, patients with ovarian, colorectal, and head and neck cancer had a higher risk of death (Table 3). In the results from the sensitivity analyses, only minimal differences in the association between class membership and overall mortality were observed between the analyses using all patients and those using only complete cases (*n* = 406; HR 1.41, 95% CI 1.03-1.92).

**Table 2:** Association between symptom class and survival using Cox proportional hazards model with four symptom classes

Covariates	Bivariate		Multivariate	
	HR (95% CI)	*P*	HR (95% CI)	*P*
Symptom class				
All low class (Ref)				
Low psychological class	1.15 (0.82-1.60)	0.418	1.25 (0.88-1.78)	0.212
High psychological class	0.91 (0.65-1.28)	0.586	1.08 (0.75-1.55)	0.674
All high class	1.40 (1.04-1.90)	**0.028**	1.63 (1.17-2.28)	**0.004**
Demographic variables				
Age	1.04 (1.03-1.06)	**<0.001**	1.03 (1.02-1.04)	**<0.001**
Sex				
Male (Ref)				
Female	0.43 (0.34-0.55)	**<0.001**	0.54 (0.38-0.77)	**0.001**
Education				
Primary (Ref)				
Secondary	0.82 (0.60-1.11)	0.195		
College/university	0.78 (0.56-1.08)	0.131		
Clinical variables				
Diagnosis				
Breast (Ref)				
Head and neck	3.53 (2.46-5.07)	**<0.001**	2.21 (1.40-3.49)	**0.001**
Colorectal	5.70 (3.98-8.17)	**<0.001**	3.49 (2.26-5.41)	**<0.001**
Ovarian	5.33 (3.68-7.71)	**<0.001**	4.72 (3.21-6.92)	**<0.001**
Number of comorbidities	1.10 (1.03-1.18)	**0.008**	1.07 (0.99-1.15)	0.079

Statistically significant *P* values are highlighted in bold.

CI, confidence interval; HR, hazard ratio; Ref, reference.

**Table 3:** Association between symptom class and survival using Cox proportional hazards model with two symptom classes

Covariates	Bivariate		Multivariate	
	HR (95% CI)	*P*	HR (95% CI)	*P*
Symptom class				
All other classes combined (Ref)				
All high class	1.39 (1.08-1.78)	**0.010**	1.48 (1.14-1.94)	**0.004**
Demographic variables				
Age	1.04 (1.03-1.06)	**<0.001**	1.03 (1.02-1.04)	**<0.001**
Sex				
Male (Ref)				
Female	0.43 (0.34-0.55)	**<0.001**	0.54 (0.38-0.76)	**<0.001**
Education				
Primary (Ref)				
Secondary	0.81 (0.60-1.09)	0.166		
College/university	0.76 (0.55-1.05)	0.096		
Clinical variables				
Diagnosis				
Breast (Ref)				
Head and neck	3.48 (2.42-4.99)	**<0.001**	2.20 (1.40-3.45)	**0.001**
Colorectal	5.50 (3.84-7.86)	**<0.001**	3.45 (2.24-5.32)	**<0.001**
Ovarian	5.07 (3.51-7.31)	**<0.001**	4.68 (3.19-6.86)	**<0.001**
Number of comorbidities	1.10 (1.03-1.18)	**0.006**	1.08 (1.00-1.16)	**0.042**

Statistically significant *P* values are highlighted in bold.

CI, confidence interval; HR, hazard ratio; Ref, reference.

## Discussion

This study explored the strength of the association between having a high number of symptoms at the time of initiation of a new treatment for cancer and overall survival of patients with breast, ovarian, colorectal, and head and neck cancer. The main finding was that patients who self-reported multiple symptoms (i.e. ‘all high’ symptom class), identified using LCA on the 13 most prevalent symptoms at the beginning of a new cancer treatment, had a significantly higher risk of death 12-15 years later, even when adjusted for well-known prognostic factors such as cancer diagnosis, comorbidities, sex, and age.

Although direct comparisons across studies are difficult because of differences in patient characteristics and PROs used, our finding is in line with a large body of previous research examining associations between symptoms and HRQoL and poorer survival. This is most commonly assessed for short-term survival (i.e. ≤5 years).^15^, ^16^, ^17^^,^^19^, ^20^, ^21^^,^^23^, ^24^, ^25^ This is the first study to assess the strength of an association between symptom class membership and long-term survival. The identification of an association between experiencing multiple co-occurring symptoms and survival in patients with cancer is important knowledge. The prevalence of multiple symptoms is high in these patients^33^ and a high symptom burden is associated with decrements in psychological well-being, functional status, work life, social life, and daily life.^34^ There has been growing attention paid to PROs as endpoints in cancer clinical trials, in addition to (progression-free) survival. For many patients, HRQoL is strongly associated with the symptoms of both the disease and its treatment. Disease control must also be interpreted in light of a meaningful benefit to symptom control.^35^ It is worth noting that the mortality risk was similar in the two intermediate subgroups (i.e. ‘high psychological’ and ‘low psychological’) and the ‘all low’ class. The two intermediate subgroups had a low-to-moderate and moderate-to-high likelihood of occurrence for the physical symptoms, respectively. This may indicate that it is less important whether the symptoms are physical or psychological symptoms than the total symptom burden.

In the present study, we adjusted for the most common sociodemographic and clinical characteristics associated with a higher risk of death. The etiology underlying the high symptom reporting among patients in the present study is still uncertain and likely multifactorial including intrinsic (e.g. genetic and epigenetic) and extrinsic (e.g. environmental exposures) factors and their intersection (e.g. lifestyle). Although no statistically significant differences were found in cancer diagnosis or disease severity (i.e. presence of metastases and treatment intent) among classes in our previous analyses, patients in the ‘all high’ subgroup may have experienced more aggressive disease, not only causing a higher symptom burden of the disease and requiring more aggressive treatment but also being associated with a higher risk of mortality. Information on disease stage is not meaningful to compare across a heterogeneous sample with several types of cancer diagnoses but there may have been differences in disease severity not adjusted for in our analyses. As depicted in Figure 1, a distinct separation in survival was apparent early in the study period, supporting differences in disease severity or aggressiveness. Independent of the mechanisms behind the symptom experience, results from the present study indicate that patients with cancer experience multiple co-occurring symptoms. In the all high symptom studied herein, patients reported more symptoms as a function of both their disease and its treatment and are at a higher risk of death.

The symptom classes in the present study are based on symptoms being reported at the beginning of a new treatment and most patients had already undergone at least one other treatment modality (e.g. surgery). However, in our previous analyses, there were no associations between the symptom class membership and type of previous treatment (e.g. CTX and surgery).^13^ We have not examined the stability of the symptom classes, meaning that patients may have shifted to a different class at a later point in time. More research is needed on long-term stability of symptom class membership or having a higher symptom burden but studies have found that symptom class membership is stable, for instance, in a large cohort of patients with colon cancer from The Netherlands.^36^

Interestingly, we found that men in the ‘all high’ class had a higher risk of dying than women in the same class despite adjustment for type of cancer diagnosis in our models (e.g. differences in prognosis between early-stage breast cancer compared with advanced stage head and neck cancer). It should be noted that the majority (69%) were female because two of the diagnostic groups consisted of females only. Our finding is supported by the literature as previous studies found men to have higher mortality rates in cancer populations.^37^^,^^38^

### Strengths and limitations

The strengths of this study are the large sample size and the long follow-up time. Limitations in this study include the heterogeneous sample (i.e. different cancer diagnoses and receipt of cancer treatments before inclusion) and the fact that we could not adjust for functional status or account for the possible bias related to stability of the classes. In addition, we lack information on subsequent cancer treatments and development of comorbidities after the identification of symptom classes using LCA, which may have influenced results. Finally, we lack information on causes of death (i.e. whether they were cancer-related).

### Conclusion

The present study is an important contribution to the literature describing an association between symptom experience and long-term survival. Our data revealed that symptom class membership appears to be an important prognostic marker identifying patients at increased risk of death many years later. This clinically meaningful and important association warrants further investigation.

## Funding

The work was supported by the 10.13039/100008730Norwegian Cancer Society [grant number 58139001].

## Ethical approval

The study was approved by the Regional Committee for Medical and Health Research Ethics (number 2009/1451), the Norwegian Directorate of Health, the privacy ombudsman at the hospital, and the institutional review board.^13^

## Disclosure

The authors have declared no conflicts of interest.
